# Spectral dependence of third-order susceptibility of Au triangular nanoplates

**DOI:** 10.1038/s41598-020-70868-4

**Published:** 2020-08-17

**Authors:** Boyi Zhang, Rodrigo Sato, Miyoko Tanaka, Yoshihiko Takeda

**Affiliations:** 1 School of Pure and Applied Sciences, University of Tsukuba, Tsukuba, Ibaraki 305-8577 Japan; 2 Center for Green Research on Energy and Environmental Materials, National Institute of Materials Science (NIMS), Tsukuba, Ibaraki 305-0003 Japan

**Keywords:** Nanophotonics and plasmonics, Nonlinear optics

## Abstract

We experimentally investigated the spectral dependence of the third-order susceptibility $\chi^{\left( 3 \right)}$ of Au triangular nanoplates in a broad wavelength region (400–1,000 nm). Complex shaped plasmonic nanoparticles provide a promising route to achieve control of their optical properties at the nanoscale. However, little is known about the effects of geometrical parameters to the optical nonlinearities and underlying mechanisms of the plasmon modes. Here, we obtained the $\chi^{\left( 3 \right)}$ of Au triangular nanoplates featuring a narrow plasmon resonance that is tunable in the visible and near-IR regions. This work demonstrates that the plasmonic triangular nanoplates simultaneously shows self-focusing and -defocusing, and saturable and reverse-saturable absorption properties at specific wavelength regions. Maximum amplitudes of real and imaginary components are − 6.8 × 10^−18^ m^2^/V^2^ at 668 nm and − 6.7 × 10^−18^ m^2^/V^2^ at 646 nm, respectively. Spectral dependence of the quantity $\chi^{\left( 3 \right)}$ enables comparison between different shaped plasmonic NPs to boost active plasmonic applications performance.

## Introduction

Metallic nanoparticles (NPs) have attracted extensive interests because of their unique plasmonic properties^1^. The light-driven collective motion of electrons within the NP is known as localized surface plasmon resonance (LSPR)^2^. This resonance can boost both linear and nonlinear optical (NLO) properties^2^. Thus, a plasmonic NP can feature large optical nonlinearity while exhibiting ultrafast response^3^. These unique properties have been applied to various nonlinear optical functionalities, such as surface-enhanced Raman scattering (SERS), photonic waveguides and ultrafast all-optical switching^1,3–5^. Among various plasmonic NPs, Au has been one of the best and common studied materials in nonlinear photonics due to its large optical nonlinearity and chemical stability^6,7^.

Triangular Au nanoplates is promising for strong local field enhancement expected at their sharp tips. Also, the LSPR of triangular nanoplates can be easily tuned from visible to near-IR regions by controlling their thickness and edge length^8^. Besides, Au nanoplates have been reported with a large wavelength dependence of nonlinear refraction and a tiny change of nonlinear absorption^9^. These results imply that Au nanoplate is a good candidate for optical switches^9,10^. However, previous studies on spherical and elongated Au NPs have shown that third-order susceptibility $\chi^{\left( 3 \right)}$ exhibits a strong wavelength-dependent dispersion at LSPR region^11,12^. Real and imaginary components of $\chi^{\left( 3 \right)}$ consist of successively positive and negative peaks. This result indicate that Au simultaneously exhibit nonlinear absorption (saturable absorption SA and reversed saturable absorption RSA) and refraction (self-focusing and self-defocusing) at different wavelengths. To clarify and understand the reported abnormal optical nonlinearity on Au nanoplates, a spectral dependent dispersion of $\chi^{\left( 3 \right)}$ is necessary. To the best of our knowledge, previously reported investigations on Au nanoplates were performed by the single-wavelength Z-scan method^9,10^. The results concentrated only on very limited wavelengths. Therefore, current scattered Z-scan results are not sufficient to give a comprehensive description to NLO properties of Au triangular plates and still require further research.

In this manuscript we investigated the spectral dependence of $\chi^{\left( 3 \right)}$ of Au triangular nanoplates by a combined analysis of pump-probe spectroscopy and spectroscopic ellipsometry. This method allows us to elucidate the complex dispersion of $\chi^{\left( 3 \right)}$ over a broad wavelength range (400–1,000 nm). Different from previous reports, real and imaginary components of $\chi^{\left( 3 \right)}$ of Au nanoplates show strong wavelength dependence in the vicinity of the plasmon modes. SA and RSA, self-focusing and self-defocusing simultaneously take place at different wavelengths. This negative and positive dispersion of $\chi^{\left( 3 \right)}$ originates from the interaction between intrinsic nonlinearity of Au and LSPR. The significant optical switching properties from previous reports only occurs at a specific wavelength region. Moreover, the maximum saturable absorption and $\chi^{\left( 3 \right)}$ locates at off-resonance wavelength, which shows a significant shift compared with linear LSPR. This result is beneficial for understanding the nonlinear behavior of plasmonic triangular nanoplates and optimizing the nonlinear plasmonic devices at desired wavelengths.

## Results and discussion

**Figure 1:**
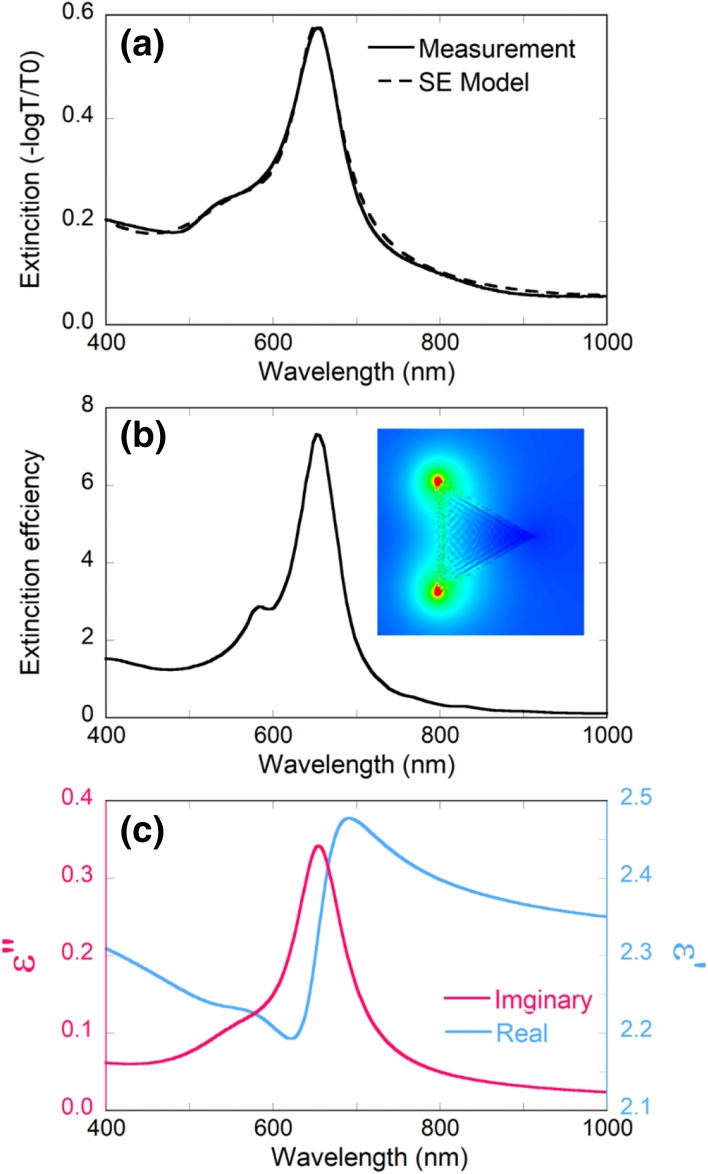
Linear optical properties of Au nanoplates: (**a**) extinction spectrum from ellipsometry measurement and ellipsometric model. (**b**) Extinction ecoefficiency from DDA simulation and corresponding dipole electric filed distribution at 655 nm. (**c**) Real (blue) and imaginary (red) components of dielectric function of nanoplates evaluated by ellipsometry.

Linear optical properties of Au nanoplates are shown in Fig. 1. Figure 1a shows the extinction spectrum from ellipsometry measurement. The spectral signatures can be understood by using discrete dipole approximation (DDA) simulation and simulated extinction efficiency is shown in Fig. 1b. The spectrum is composed of a strong absorption band at 655 nm. This peak originates from the dipole plasmon mode of nanoplates corresponding to electric filed distribution shown on the right side of Fig. 1b^13^. A large field enhancement is expected to appear at the tips. At shorter wavelengths, another absorption peak with lower amplitude can be ascribed to the quadrupole plasmon mode^14^. Compared with DDA simulation, quadrupole mode absorption from experiments gets broader and shifts to 540 nm. This indicates the contribution from minimal impurities (spherical NPs). Interband transitions from 4d band to 5sp band contributes to the absorption band below 540 nm^15^. In all, consistence between DDA simulation and experimental results suggests that Au nanoplates are well dispersed in PVA and can be considered as the ensemble of single Au nanoplates. The Au/PVA composite behaves as an ensemble of single nanoplates without aggregation. Dielectric function of the Au nanoplates/PVA composite was evaluated by ellipsometry and plotted in Fig. 1c. The complex dielectric function was described by three Lorentz oscillators and fitted to ellipsometric data. The mean squared error (MSE) of our ellipsometric model was 17.0, which indicates a good fitting quality to evaluate the optical properties of the composite^16^. This minimized MSE allows to consider the permittivity in Fig. 1c adequate to extract the third-order susceptibility from pump-probe measurements.

**Figure 2:**
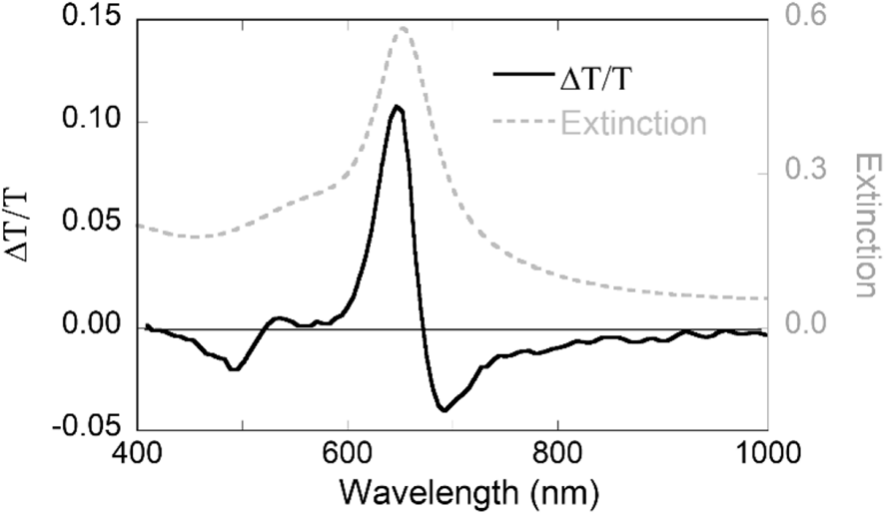
Transient transmission changes of Au nanoplates composite measured by ultrafast pump-probe spectroscopy. The grey dashed line represents the linear extinction spectrum extracted from Fig. 4a. for comparison.

To clarify the NLO properties, ∆T/T of the composites were measured by ultrafast pump and probe spectroscopy and shown in Fig. 2. This transient SA and RSA reflect the laser-induced modulation on NLO properties. The spectrum consists of several successively SA and RSA peaks. The small RSA locates below 450 nm originating from the interband transitions of Au and shares a similar line shape compared with bulk Au^12^. The strongest dipole polarization contributes to the sharp SA located at 647 nm and follows a broad RSA extending towards infrared region. As a comparison, the linear extinction was taken from Fig. 1a and plotted together with ∆T/T. Note that, the wavelength of maximum ∆T/T shows a blue shift compared with linear LSPR. Following the wavelength of linear LSPR, the measurement of nonlinearity will return a weaker value or even opposite sign of nonlinearity.

**Figure 3:**
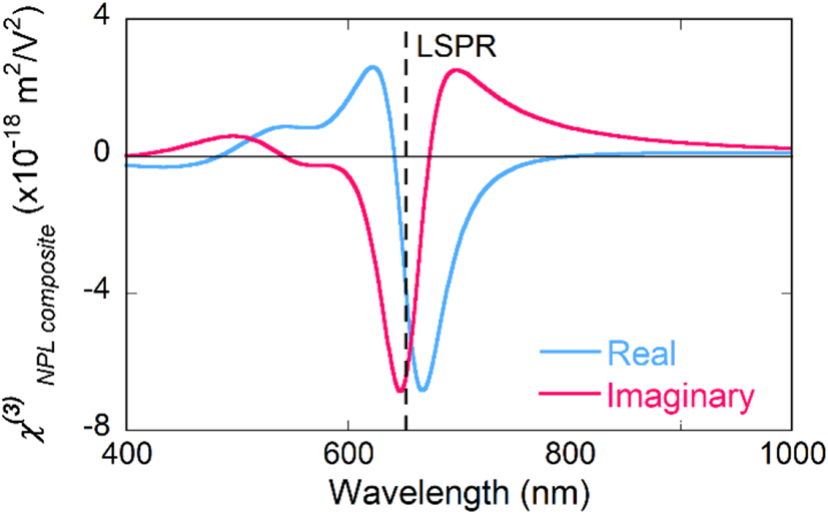
Real (blue) and imaginary (blue) components of third-order susceptibility of Au triangular nanoplates embed in PVA matrix. The straight dashed line represents the wavelength of linear LSPR.

By the combined analysis of ∆T/T and the dielectric function from ellipsometry, real and imaginary components of $\chi^{\left( 3 \right)}$ can be derived by^17^:

1 $$\Delta {\upvarepsilon }\left( {\omega_{probe} } \right) = \frac{3}{4}\chi_{{Au{ }NPL}}^{\left( 3 \right)} \left( {\omega_{probe} } \right)I,$$

where $\frac{3}{4}$ is the K factor for intensity dependent refractive index^18^ and *I* the pump laser intensity. $\Delta {\upvarepsilon }$ stands for the dielectric function modulation for Au/PVA composite and calculated from the difference of dielectric function at excited state (${\upvarepsilon } + \Delta {\upvarepsilon }$) and steady state (${\upvarepsilon }$). ${\upvarepsilon } + \Delta {\upvarepsilon }$ was evaluated by fitting the linear SE model to nonlinear transmission ${\text{ T}} + \Delta {\text{T}}$ as previously reported. Structural information (thickness, surface roughness and so on) remained same and only dielectric function (parameters of Lorentz oscillators) was modified in this process. The evaluated real and imaginary components of $\chi^{\left( 3 \right)}$ were plotted in Fig. 3. Both components have shown distinctive dispersions consisted of positive and negative peaks, which indicates the nonlinear absorption and refraction, respectively. The maximum amplitude of $\chi^{\left( 3 \right)^{\prime\prime}}$ locates at 646 nm with a magnitude of − 6.7 × 10^–18^ m^2^/V^2^. This peak reflects the SA observed in ∆T/T spectrum. At wings, two positive band indicates the interband contribution at lower wavelength and LSPR contribution towards further IR region. For real components, maximum amplitude of $\chi^{\left( 3 \right)^{\prime}}$ appears at 668 nm with a magnitude of -6.8 × 10^–18^ m^2^/V^2^. One can note that $\chi^{\left( 3 \right)}$ of nanoplates did not show several orders intense enhancement compared with Au spheres^12^. One possible assumption is that the tips of fabricated nanoplates is not as sharp as simulation. Another assumption could be due to the average local field among whole nanoplates is not as strong as it at the sharp tips, which is usually considered by most theoretical calculations^19^. Also, linear LSPR peak (straight dashed line) locates at 655 nm, in between of the maximum aptitude of real and imaginary components. The off-resonance location have been also been observed in Au nanorods^20^. For spherical nanoparticle, both linear and nonlinear peak locates around 540 nm. However, with tuning the LSPR towards infrared region by geometry modification, the nonlinear LSPR starts to show this wavelength shift compared linear one. This off-resonance shift could be significant when taking use of nanoparticles around LSPR wavelength.

Shown in Fig. 3, $\chi^{\left( 3 \right)}$ of nanoplates displays a positive–negative dispersion and a large wavelength dependence around LSPR. Real and imaginary components have similar maximum amplitudes at different wavelength, which indicates that Au nanoplates own considerable nonlinear refraction and absorption properties simultaneously. Notice that due to the differential nature describing by Kronig–Kramers relationship^21^, the maximum amplitude of the real (imaginary) component accompanies with a crossover of the imaginary (real) component at the same wavelength. Thus, functionalities taking use of nonlinear refraction or absorption should be specified at a certain wavelength. These spectral signatures originate from interactions between intrinsic Au nonlinearity and LSPR^12,22,23^. The intrinsic nonlinearity of Au has already been well investigated and described by laser-induced electron distribution change^22^. While exhibiting same intrinsic nonlinearity, modification of geometries results in the different polarization of LSPR^14^. The key feature of nanoplates is the near-infrared dipole polarization and higher order plasmon modes close to interband region. Tuning LSPR towards IR region with intense local field enhancement can be easily achieved by controlling geometry of nanoplates rather than NPs. Another candidate for IR applications is elongated NPs, such as nanorods or nanobipyramids^11,19,20^. Those particles have two strong dipole polarization simultaneously at different wavelength. The transverse mode is similar to spherical NPs and the longitudinal mode locates at IR region. Compared with elongated NPs, nanoplates have the advantage from higher ratio of surface to bulk atoms and the ability to support multipole plasmon modes, such as higher-order eigenmodes or out-of-plane mode. In all, nanoplates supports LSPR modes at longer wavelength compared with spherical nanoparticles with complex valued and wavelength dependent $\chi^{\left( 3 \right)}$. $\chi^{\left( 3 \right)}$ of nanoplates shows a significant shift compared with linear LSPR peak.

Having understood with the dispersion of the real and imaginary components of $\chi^{\left( 3 \right)}$, abnormal results reported by Li et al*.*^10^ could be clarified. Direct comparison of the intensity of $\chi^{\left( 3 \right)}$ at same wavelength is not feasible due to different LSPR modes exhibiting from different sizes of nanoplates. Li’s group has measured a nanoplate solution with the edge length of 90 nm. The LSPR locates around 1,200 nm and the peak is broad due to the impurities and broad size distribution. The reported values of $\chi^{\left( 3 \right)}$ are (6.37 + i1.21) × 10^–13^ esu at 800 nm and (− 125 + i1.03) × 10^–13^ esu at 1,240 nm, respectively. (The reported value of $\chi^{{\left( 3 \right){^{\prime}}}}$ at 1,240 nm is 125 × 10^–13^ esu. However, we believe that the authors have applied an incorrect conversion formula in converting closed aperture measurement to $\chi^{{\left( 3 \right){^{\prime}}}}$. The positive–negative dispersion in closed-aperture measurements should be converted to a negative sign of $\chi^{{\left( 3 \right){^{\prime}}}}$.) The huge $\chi^{{\left( 3 \right){^{\prime}}}}$ at 1,240 nm was ascribed to the dipole resonance of nanoplates while $\chi^{{\left( 3 \right)^{{^{\prime\prime}}} }}$ is not sensitive to wavelength. First of all, clarification of the wavelength regime of their measurement is necessary. They simply assumed that the peak of T and ∆T/T locate at the same wavelength. However, the open-aperture Z-scan measurement at 1,240 nm shows negative sign of ∆T/T, which correspond. Shown in Fig. 3, the linear LSPR locates at a longer wavelength than SA peak of ∆T/T, where ∆T/T decreases rapidly and shows a crossover from SA to RSA. The possible Z-scan measurement region was marked together with Fig. 3 and can be found as Supplementary Fig. S1 online. Next, the corresponding spectral dependence at this region can be achieved. As shown in Fig. 3, the maximum amplitude of $\chi^{{\left( 3 \right){^{\prime}}}}$ locates around this crossover while $\chi^{{\left( 3 \right){^{\prime\prime}}}}$ increase rapidly with a sign reversion form negative (SA) to positive (RSA). Hence, we can summarize that, Li et al*.*, have conducted their measurement at linear LSPR wavelength, several tenth nanometers red-shifted compared with SA of ∆T/T. Consequently, a strong wavelength dependence of nonlinear refraction and a tiny change on nonlinear absorption was observed. Limited by the scattered data at 800 nm and 1,240 nm, they concluded that Au nanoplates as a promising candidate for broadband optical-switching. Shown in Fig. 3, this conclusion is only valid at a specific wavelength region. Finally, indicated by this comparison with scattered Z-scan measurements, the dispersion of $\chi^{\left( 3 \right)}$ can be crucially important to comprehensively interpret the NLO properties. The strong spectral dependence and off-resonance located $\chi^{\left( 3 \right)}$ provides insight into the design of plasmonic metamaterials at an optimized wavelength.

## Conclusion

We investigated the dispersion of $\chi^{\left( 3 \right)}$ of Au nanoplates/PVA composite in a broad wavelength region (400–1,000 nm) through a combined analysis of spectroscopic ellipsometry and pump-probe spectroscopy. $\chi^{\left( 3 \right)}$ of nanoplates shows a complex wavelength dependence resulting from intrinsic nonlinearity of Au and plasmon modes. Both real and imaginary components of $\chi^{\left( 3 \right)}$ have a strong negative peak around LSPR wavelength and two opposite band at the wings. Maximum amplitudes of real and imaginary components are − 6.8 × 10^–18^ m^2^/V^2^ at 668 nm and − 6.7 × 10^–18^ m^2^/V^2^ at 646 nm, respectively. In particular, the real (imaginary) component shows a significant shift towards longer (shorter) wavelength compared with linear LSPR. The dispersion offers a comprehensive understanding compared with previous reported nonlinearity of Au nanoplates. This result indicates the importance of the dispersion of the quantity $\chi^{\left( 3 \right)}$ to optimize the nonlinear absorption or refraction properties at a desired wavelength.

## Methods

**Figure 4:**
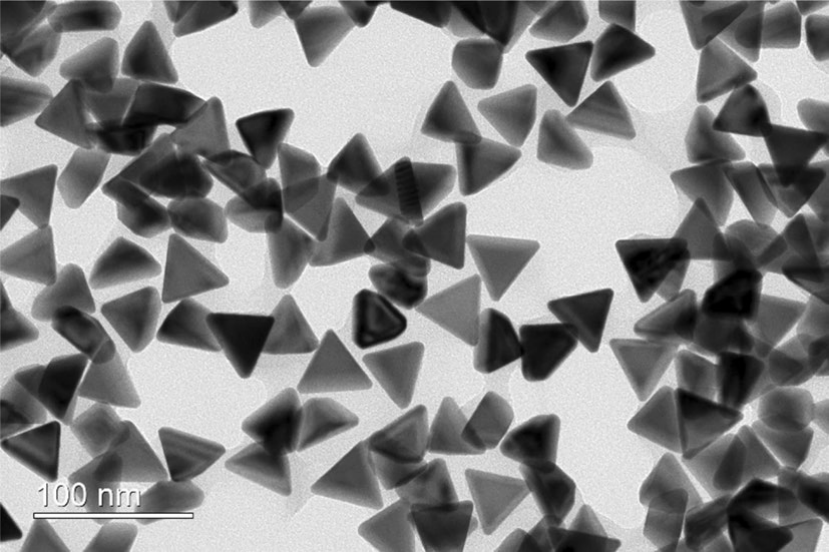
TEM image of Au nanoplates with an edge length of 45 nm.

Au triangular nanoplates dispersed in water with ~ 45 nm edge length were bought from Dai Nippon Toryo Co., Ltd. The TEM measurements was carried and shown in Fig. 4. TEM observation is performed using JEM-2100F(JEOL) and operating at 200 kV. The solution of Au nanoplates was diluted with equal amount of ethanol, treated with ultrasonication for 15 min, and then dropped onto a TEM carbon grid. Those NPs were used without any further treatment and had been embedded in a poly(vinyl alcohol)(PVA) matrix by a spin coating method as reported by Sato *et al*^24^. Nanoplates were separated from the mother liquid by centrifugation. The precipitated NPs was redispersed into PVA solution (20 g/l). 15 μl of this NPs/PVA suspension was then spin-coated (800 RPM, 10 min) on an amorphous silica glass substrate. The substrates were precleaned by an ozone cleaner before spin coating. The computational simulations of the extinction efficiency and electric field distributions were performed by DDA method using DDSCAT^26^. The edge length of the Au NPLs was set to be 45 nm and the thickness was chosen as 30 nm to fit the LSPR wavelength. The refractive index of the medium was approximately 1.5 at all wavelength (extracted from a pure PVA thin film by spectroscopic ellipsometry) and dielectric function of Au was taken from Palik’s report^27^. All the simulations were performed with an incident filed perpendicular to the triangular plane of the nanoplates so that only in-plane polarization mode was excited.

Linear optical properties and thicknesses of those Au/PVA composites were measured by a variable angle spectroscopic ellipsometry (SE) (J.A. Woollam, VASE). Parameters including phase and polarization changes of reflected light were collected at incident angles of 50°, 60°, and 70° from 300 to 1,000 nm. Extinction (− log (T/T_0_)) of the composite was measured at incident angle of 90° from 300 to 1,000 nm, where T represents the transmittance of nanoplate composite on silica substrates and T_0_ is the transmittance of baseline (air). Several Lorentz oscillators were applied to fit the reflection and extinction data for the evaluation of in-plane dielectric function *ε*^28^. The parameters of each Lorentz oscillator can be found as Supplementary S2 online. The thickness of the composite was evaluated as 590 nm. The nonlinear response of composites was measured by a custom-made pump and probe spectroscopy. The scheme of pump and probe spectroscopy can be found as Supplementary Fig. S3 online. The quantities measured were transient transmission changes (∆T/T), which were defined as the ration between transmitted light with and without laser excitation. Fundamental laser source was supplied by a Ti:sapphire regenerative amplifier (Spitfire, Spectra-Physics) seeded with an oscillator (Mai Tai, Spectra-Physics) and pumped by a diode-pumped laser (Empower, Spectra-Physics). The fundamental beam with an output pulse of 130 fs at 800 nm and 1 kHz repetition was divided into two portions: pump beam and probe beam. Pump beam at 400 nm was generated by a BBO crystal through second harmonic generation. Also, the repetition rate of pump beam was converted to 0.5 kHz by an optical chopper. The sample was illuminated with a peak intensity of 1 GW/cm^2^. The supercontinuum probe beam was generated using Al_2_O_3_ and YAG crystals for visible (400–800 nm) and near infrared region (800–1,000 nm), respectively. Group velocity correction of the raw data was done according to chirping effect using the Kerr gate technique^29^.

## Supplementary information

Supplementary information.

## References

[1] Kauranen M, Zayats AV (2012). Nonlinear plasmonics. Nat. Photonics.

[2] Hutter E, Fendler JH (2004). Exploitation of localized surface plasmon resonance. Adv. Mater..

[3] Ren M (2011). Nanostructured plasmonic medium for terahertz bandwidth all-optical switching. Adv. Mater..

[4] Tame MS (2013). Quantum plasmonics. Nat. Phys..

[5] Pang C (2018). Lithium niobate crystal with embedded au nanoparticles: a new saturable absorber for efficient mode-locking of ultrafast laser pulses at 1 µm. Adv. Opt. Mater..

[6] Ghosh SK, Pal T (2007). Interparticle coupling effect on the surface plasmon resonance of gold nanoparticles: from theory to applications. Chem. Rev..

[7] Wu L, Chu HS, Koh WS, Li EP (2010). Highly sensitive graphene biosensors based on surface plasmon resonance. Opt. Express.

[8] Chen L (2016). Halide-free synthesis of Au nanoplates and monitoring the shape evolution process through a marker experiment. J. Mater. Chem. C.

[9] Chen Z (2013). Dipole plasmon resonance induced large third-order optical nonlinearity of Au triangular nanoprism in infrared region. Opt. Express.

[10] Li Z (2013). Ultrafast third-order optical nonlinearity in au triangular nanoprism with strong dipole and quadrupole plasmon resonance. J. Phys. Chem. C.

[11] Baida H (2011). Ultrafast nonlinear optical response of a single gold nanorod near its surface plasmon resonance. Phys. Rev. Lett..

[12] Zhang B (2019). Spectral dependence of the third-order optical susceptibility of Au nanostructures: experiments and first-principles calculations. Phys. Rev. B.

[13] Yang P, Portalès H, Pileni MP (2009). Identification of multipolar surface plasmon resonances in triangular silver nanoprisms with very high aspect ratios using the DDA method. J. Phys. Chem. C.

[14] Keast VJ (2016). Higher order plasmonic modes excited in Ag triangular nanoplates by an electron beam. Plasmonics.

[15] Smith DD (1999). z-scan measurement of the nonlinear absorption of a thin gold film. J. Appl. Phys..

[16] Jiang HQ, Wei Q, Cao QX, Yao X (2008). Spectroscopic ellipsometry characterization of TiO2 thin films prepared by the sol-gel method. Ceram. Int..

[17] Sato R, Ohnuma M, Oyoshi K, Takeda Y (2014). Experimental investigation of nonlinear optical properties of Ag nanoparticles: effects of size quantization. Phys. Rev. B.

[18] Butcher PN, Cotter D (1990). The Elements of Nonlinear Optics.

[19] Liu M, Guyot-Sionnest P, Lee T-W, Gray SK (2007). Optical properties of rodlike and bipyramidal gold nanoparticles from three-dimensional computations. Phys. Rev. B.

[20] Sato R, Henzie J, Rong H, Naito M, Takeda Y (2019). Enhancement of the complex third-order nonlinear optical susceptibility in Au nanorods. Opt. Express.

[21] Kador L (1995). Kramers-Kronig relations in nonlinear optics. Appl. Phys. Lett..

[22] Marini A (2013). Ultrafast nonlinear dynamics of surface plasmon polaritons in gold nanowires due to the intrinsic nonlinearity of metals. New J. Phys..

[23] Stoll T, Maioli P, Crut A, Del Fatti N, Vallée F (2014). Advances in femto-nano-optics: ultrafast nonlinearity of metal nanoparticles. Eur. Phys. J. B.

[24] Sato R, Ishii S, Nagao T, Naito M, Takeda Y (2018). Broadband plasmon resonance enhanced third-order optical nonlinearity in refractory titanium nitride nanostructures. ACS Photonics.

[25] Onodera H (2002). Microstructures and magnetic properties of Co–Al–O granular thin films. J. Appl. Phys..

[26] Draine BT, Flatau PJ (1994). Discrete-dipole approximation for scattering calculations. J. Opt. Soc. Am. A.

[27] Palik ED (1998). Handbook of Optical Constants of Solids.

[28] Fujiwara H (2007). Spectroscopic Ellipsometry. Spectroscopic Ellipsometry: Principles and Applications.

[29] Tan W, Liu H, Si J, Hou X (2008). Control of the gated spectra with narrow bandwidth from a supercontinuum using ultrafast optical Kerr gate of bismuth glass. Appl. Phys. Lett..

