# Supplementary material for: Spectral dependence of third-order susceptibility of Au triangular nanoplates
Source: Sci Rep. 2020 Aug 17;10:13855. doi: 10.1038/s41598-020-70868-4 (PMC7431854; doi:10.1038/s41598-020-70868-4)
Supplement: Supplementary file 1 — Supplementary information. [file 41598_2020_70868_MOESM1_ESM.docx]

**Spectral dependence of third-order susceptibility of Au triangular nanoplates**

Boyi Zhang^1,2,*^, Rodrigo Sato^2^, Miyoko Tanaka^2^, Yoshihiko Takeda^1,2,†^

^1^School of Pure and Applied Sciences, University of Tsukuba, Tsukuba, Ibaraki 305-8577, Japan

^2^Center for Green Research on Energy and Environmental Materials, National Institute of Materials Science (NIMS), Tsukuba, Ibaraki,305-0003, Japan

^*^zhang.boyi@nims.go.jp

**^†^**takeda.yoshihiko@nims.go.jp

**Supplementary Material**

**S1. Comparison with single Z-scan measurements**

In order to help understand the comparison between single-wavelength Z-scan reports and our dispersion, the Z-scan measurements [DOI: 10.1021/jp403308k] was marked in Figure 3 as below:


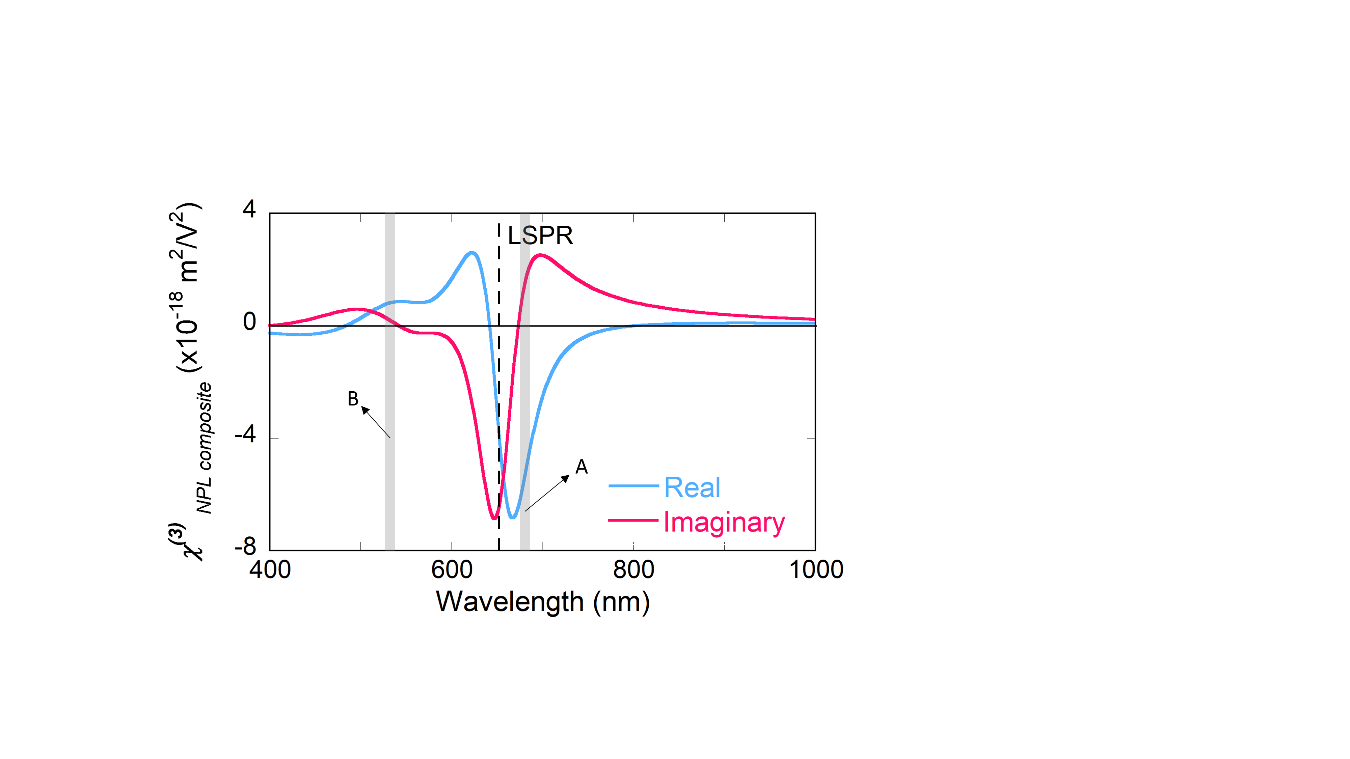
Fig S1. The comparison with previous Z-scan reports, which is marked as grey region A and B. The dispersion of $\chi^{\left( 3 \right)}$ in this manuscript is represented by blue and red lines taken from manuscript. Black dotted line denotes LSPR wavelength.

Note that the wavelength of two reports cannot be directly compared since the particle size is different. We compare the relative wavelength instead: near LSPR (A) and off-resonance wavelength (B). One should also take care that the linear LSPR wavelength (black dotted line) won’t overlap with maximum amplitude of $\chi^{\left( 3 \right)}$.

Li *et al.* $\chi^{\left( 3 \right)}$ reported (-125+i1.03) ×10^-13^ esu at 1240 nm near LSPR, which corresponding to region A in Fig. 2. At this wavelength, a large negative real component with a weak positive imaginary component was observed. Also, they tested off-resonance wavelength at 800 nm with a value of (6.37+i1.21) ×10^-13^ esu, which corresponding to region B. Here they observed both components turn to a weak positive value. Thus, they concluded that Au nanoplates owns a strong wavelength dependence of nonlinear refraction and a tiny change on nonlinear absorption, which is promising for broadband optical-switching.

Shown in our dispersion data, the reported Z-scan results are consistent at different wavelength. However, the two scattered measurements led to an inappropriate conclusion. As reported by our results, real and imaginary components of $\chi^{\left( 3 \right)}$ shows a complex dispersion and change dramatically around LSPR.

**S2. Ellipsometry model**

$\boldsymbol{\chi}^{\boldsymbol{(3)}}$ is proportional to the change of dielectric function. And the dielectric function at steady state and excited state were fitted by spectroscopic ellipsometry. We applied the Lorentz oscillators to fit the dielectric function.

$$\varepsilon_{NPL}=\sum_{1}^{3} \varepsilon_{Lorentz}=\sum_{1}^{3} \frac{{Amp}_{n}{Br}_{n}{En}_{n}}{{En}_{n}^{2}-E^{2}-i\cdot E{Br}_{n}}$$

This is a version of the classic Lorentz oscillator model, where ${Amp}_{n}$ approximately equals $\varepsilon_{2}$ at its peak value, ${Br}_{n}$ is approximately the full width at half-maximum (FWHM), ${En}_{n}$ represents the energy position and $E$ represents the wavelength (photon energy).

Following your suggestions, the parameters of each oscillators at steady state (Table I) and excited state (Table II) were listed below and added to supplementary information S3:

**Table I. Fitting parameters of the oscillators at steady state**

| Lorentz |  | Amp | Br (eV) | En (eV) |  |
| --- | --- | --- | --- | --- | --- |
| 1  2  3 |  | 0.290  0.058  0.059 | 0.204  0.597  2.935 | 1.89  2.21  3.89 |  |

**Table II. Fitting parameters of the oscillators at excited state**

| Lorentz |  | Amp | Br (eV) | En (eV) |  |
| --- | --- | --- | --- | --- | --- |
| 1  2  3 |  | 0.264  0.060  0.059 | 0.218  0.691  2.780 | 1.89  2.21  3.89 |  |

**S3. Pump and probe spectroscopy**


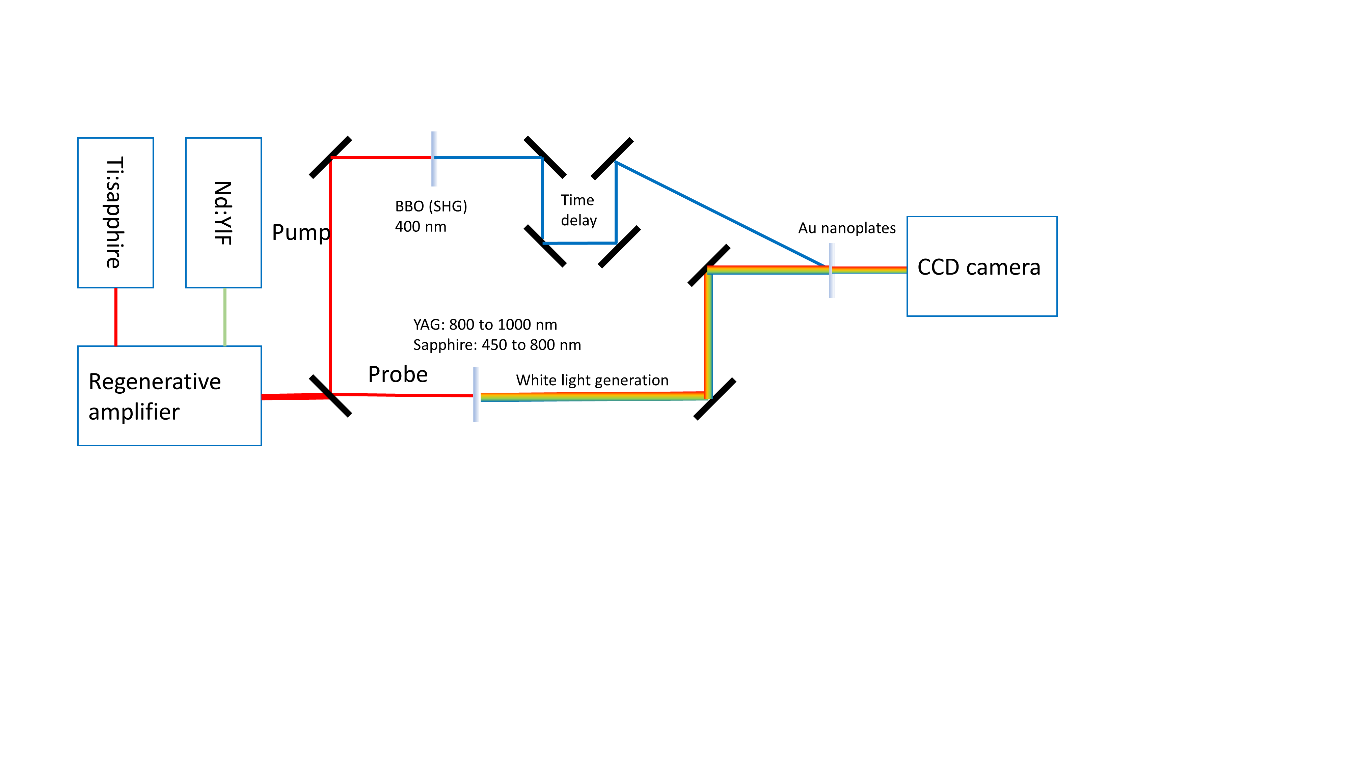
We use a custom-made femtosecond pump and probe spectroscopy. The scheme of our system is shown in Fig. S3 as below:

Fig. S3. Scheme of pump and probe spectroscopy. This image is created using Microsoft PowerPoint.(URL: www.office.com Version: Office 365)

The quantities measured were transient transmission changes (∆T/T), which were defined as the ration between transmitted light with and without laser excitation. Fundamental laser source was supplied by a Ti:sapphire regenerative amplifier (Spitfire, Spectra-Physics) seeded with an oscillator (Mai Tai, Spectra-Physics) and pumped by a diode-pumped laser (Empower, Spectra-Physics). The fundamental beam with an output pulse of 130 fs at 800 nm and 1kHz repetition was divided into two portions: pump beam and probe beam. Pump beam at 400 nm was generated by a BBO crystal through second harmonic generation. Also, the repetition rate of pump beam was converted to 0.5 kHz by an optical chopper. The sample was illuminated with a peak intensity of 1 GW/cm^2^. The supercontinuum probe beam was generated using Al_2_O_3_ and YAG crystals for visible (400 – 800 nm) and near infrared region (800 – 1000 nm), respectively. Group velocity correction of the raw data was done according to chirping effect using the Kerr gate technique.
